# Supplementary material for: A systems immunology analysis of Alzheimer's disease reveals an age‐ and environmental exposure‐independent disturbance in B cell maturation
Source: Alzheimers Dement. 2025 Nov 25;21(11):e70952. doi: 10.1002/alz.70952 (PMC12645157; doi:10.1002/alz.70952)

**Supplementary material**

**Supplementary Figure 1. Distortions in the immunological relationships present in Alzheimer’s disease patients.** The correlation coefficients were calculated between each pair of AD-associated immunological parameters. Correlations present in the healthy population (top-right) are shown, with colour indicating the strength and direction of the observed correlation. Deviations present in AD patients are shown in the bottom right, with colour and size indicating the strength and direction of correlation changes, from the baseline set in healthy individuals. No value indicates preservation of the relationship observed in healthy individuals.

**
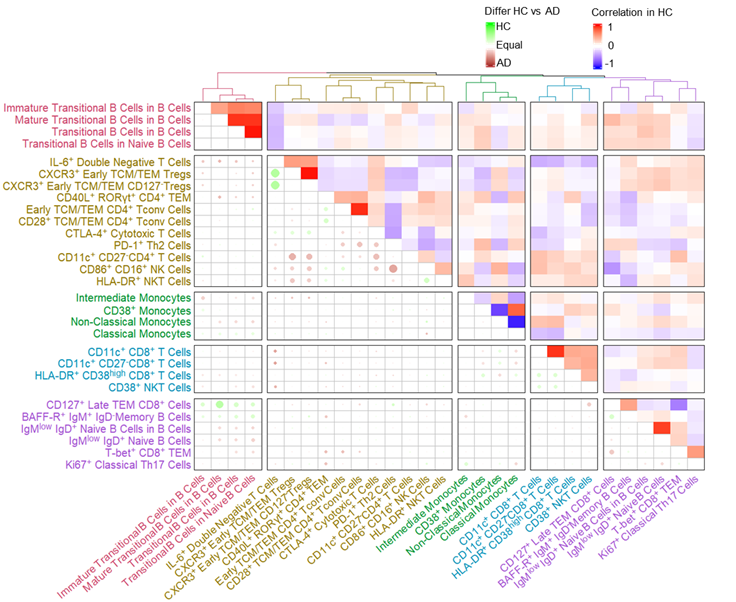
**

**Supplementary Figure 2. No major genotype associations of immunological changes in Alzheimer’s disease.** AD patients were segregated based on APOE‐ε4 genotype (102 carriers, 82 non-carriers), and assessed for genotype-dependent immune phenotype effects. Group differences were evaluated by a two-sided Mann–Whitney U test and p values were adjusted for multiple comparisons using the Benjamini–Hochberg procedure.


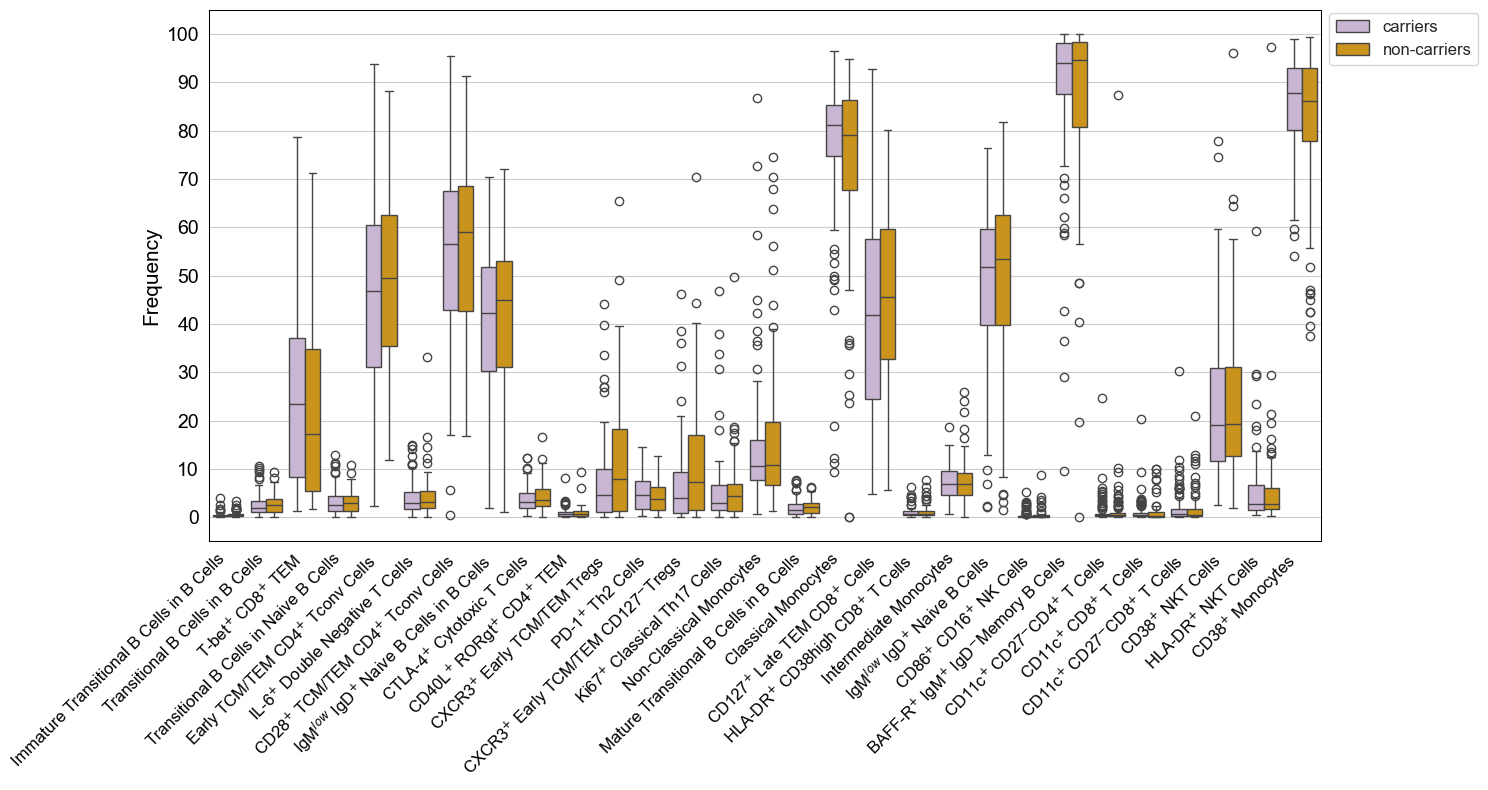


**Supplementary Figure 3. Immature transitional B cells associated with disease severity in Alzheimer’s disease.** **A)** Forest plot of Tobit regression estimating the association between the frequency of highly-associated cell populations and Mini-Mental State Examination (MMSE) scores, adjusted by sex and age. **B)** Frequency of immature transitional B cells within the B cell population for AD patients (n=152) and healthy individuals (HC) (n=91), classified based on MMSE category. **C)** Association testing between of immature transitional B cell within B cells and *APOE* ε4–disease status. Violin plots of immature transitional B cell within B cells among four groups: non-carriers (HC), non-carriers (AD), carriers (HC), and carriers (AD). Statistical differences were assessed using the Kruskal–Wallis test followed by Dunn’s post hoc FDR-BH corrections. Each dot represents a patient/control and each colour represents a condition. **D)** Linear regression models adjusted for age and sex for correlation between immature transitional B cell frequencies within total B cells and amyloid-β or Tau biomarkers. In AD patients CSF Aβ42/t-Tau ratios, CSF Aβ42/p-Tau181 ratios, and plasma Tau217 levels. In healthy controls, plasma p-Tau217 levels.


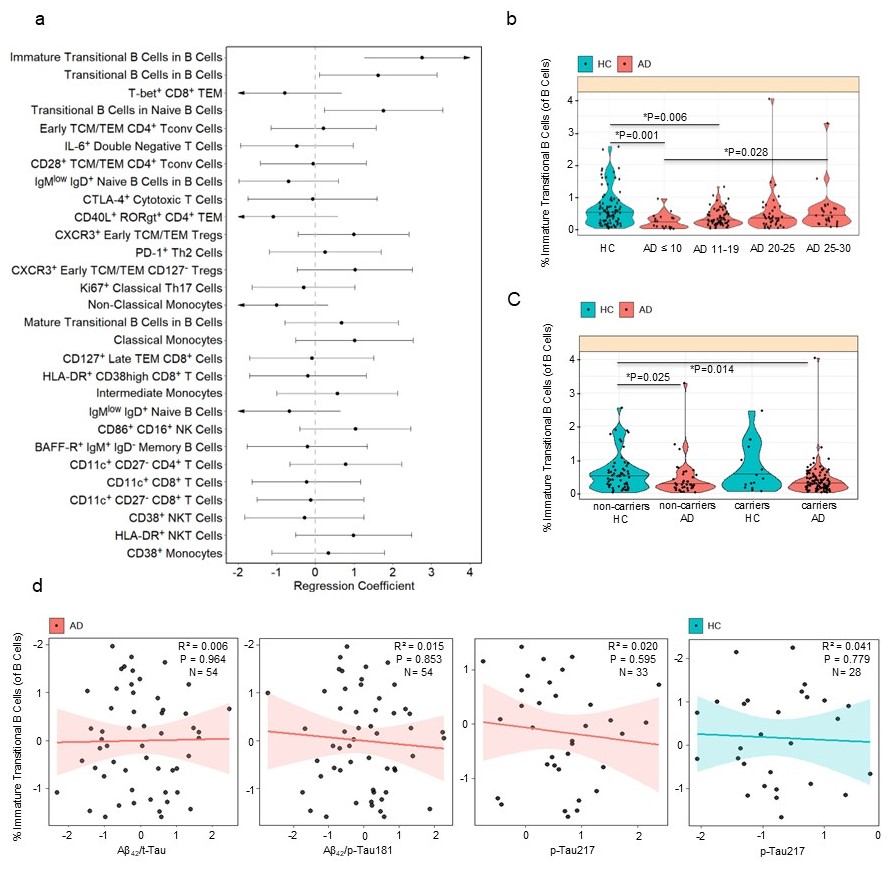

Supplement: Supplementary file 2 — Supporting Information [file ALZ-21-e70952-s004.docx]
